# Chicago City Hospital

**Published:** 1859-09

**Authors:** 


### CHICAGO CITY HOSPITAL.

This institution is now open for the reception of patients, under the care of the medical board already noticed in the Journal. The terms of service of Drs. Brainard and Miller will commence Oct. 1 of each year, and continue four months. Patients from a distance can receive the benefits of the institution by paying board, without further charge.

It is a source of no little gratification to us to see this fine building put at length to the use for which it was intended, and so perfectly adapted. The opening of it has been effected in face of a strong opposition coming from the homœopathists, and a part of the medical staff of the so-called Mercy Hospital, who in this matter have co-operated with a zeal and perseverance indicating a full understanding.

We have already had occasion to state the terms upon which that commencement opened, and the dishonest way in which those who furnished it had been deprived of attendance in it. This, since the opening of the City Hospital, has become a matter of little consequence, as the city and county poor will soon have a building adapted to their wants, and the sooner the Mercy Hospital is closed the better it will be for all parties. The number of cases of typhoid fever, etc., occurring among the students who have visited it sufficiently indicates the condition of its inmates. It is but justice, however, to state that those who have charge of it, even if more accustomed to the proper conducting of a hospital than they are supposed to be, could but imperfectly remedy the evils experienced, as the building was originally intended for other purposes, and is not adapted to its present uses.

How the physicians who are connected with it, and who have made every effort to defeat the opening of the City Hospital, in concert with irregulars, by repeated false publications in the daily papers and otherwise, can justify themselves, we are at a loss to conjecture. Certain we are that the enlightened medical profession of the North-West will not in any manner endorse such a proceeding.

